# Effects of heme oxygenase-1 on induction and development of chemically induced squamous cell carcinoma in mice

**DOI:** 10.1016/j.freeradbiomed.2011.07.025

**Published:** 2011-11-01

**Authors:** Halina Was, Malgorzata Sokolowska, Aleksandra Sierpniowska, Paweł Dominik, Klaudia Skrzypek, Bozena Lackowska, Antoni Pratnicki, Anna Grochot-Przeczek, Hevidar Taha, Jerzy Kotlinowski, Magdalena Kozakowska, Andrzej Mazan, Witold Nowak, Lucie Muchova, Libor Vitek, Anna Ratajska, Jozef Dulak, Alicja Jozkowicz

**Affiliations:** a Department of Medical Biotechnology, Faculty of Biochemistry, Biophysics, and Biotechnology, Jagiellonian University, Gronostajowa 7, 30-387 Krakow, Poland; b Department of Biophysics, Faculty of Biochemistry, Biophysics, and Biotechnology, Jagiellonian University, 30–387 Krakow, Poland; c Department of Pathology, Oncology Center, Krakow, Poland; d Department of Pathological Anatomy, Medical University of Warsaw, Warsaw, Poland; e Charles University, Prague, Czech Republic

**Keywords:** Heme oxygenase-1, Carcinogenesis, Squamous cell carcinoma, DMBA, Inflammation, Oxidative stress, Free radicals

## Abstract

Heme oxygenase-1 (HO-1) is an antioxidative and cytoprotective enzyme, which may protect neoplastic cells against anticancer therapies, thereby promoting the progression of growing tumors. Our aim was to investigate the role of HO-1 in cancer induction. Experiments were performed in HO-1^+/+^, HO-1^+/−^, and HO-1^−/−^ mice subjected to chemical induction of squamous cell carcinoma with 7,12-dimethylbenz[*a*]anthracene and phorbol 12-myristate 13-acetate. Measurements of cytoprotective genes in the livers evidenced systemic oxidative stress in the mice of all the HO-1 genotypes. Carcinogen-induced lesions appeared earlier in HO-1^−/−^ and HO-1^+/−^ than in wild-type animals. They also contained much higher concentrations of vascular endothelial growth factor and keratinocyte chemoattractant, but lower levels of tumor necrosis factor-α and interleukin-12. Furthermore, tumors grew much larger in HO-1 knockouts than in the other groups, which was accompanied by an increased rate of animal mortality. However, pathomorphological analysis indicated that HO-1^−/−^ lesions were mainly large but benign papillomas. In contrast, in mice expressing HO-1, most lesions displayed dysplastic features and developed to invasive carcinoma. Thus, HO-1 may protect healthy tissues against carcinogen-induced injury, but in already growing tumors it seems to favor their progression toward more malignant forms.

## Highlights

► We investigate the role of HO-1 in development of squamous cell carcinoma in mice. ► HO-1 deficient mice are more vulnerable to the DMBA/PMA-induced skin injury. ► Lack of HO-1 results in the development of large, but benign papillomas. ► HO-1 expression facilitates transformation of growing tumors to malignant carcinoma. ► HO-1 expression promotes the c-myc mediated transformation of primary fibroblasts.

Heme oxygenases (HOs) are the rate-limiting enzymes in catabolism of heme that convert it to the biologically active products carbon monoxide (CO), biliverdin, and ferrous iron [1]. Two distinct variants of HOs have been described in humans and rodents: HO-2, present at high levels in the brain, testes, or endothelial cells [2], and HO-1, which is strongly induced in many cell types by heme, heavy metals, inflammatory cytokines, UV irradiation, and reactive oxygen species [3]. Expression of HO-1 is usually higher in cancer cells than in surrounding healthy tissues, as shown for lymphosarcoma, prostate cancers, brain tumors, adenocarcinoma, hepatoma, squamous carcinoma, glioblastoma, melanoma, Kaposi sarcoma, or pancreatic carcinoma [4–12]. HO-1 can be directly induced by some oncogenes, such as viral G-protein-coupled receptor encoded by Kaposi sarcoma-associated herpes virus or BCR/ABL fusion kinase [10]. The potential meaning of such induction for oncogenesis has not been investigated yet.

Expression of HO-1 in tumors can be further elevated in response to chemo-, radio-, or photodynamic therapies, possibly as a result of oxidative stress [10,14,15]. There are several lines of evidence showing that HO-1 exerts potent and comprehensive protumoral effects in growing tumors. Its upregulation improves the survival of various tumor cell lines exposed to stressful agents both in vitro [8,16–20] and in vivo [21,22]. It may also influence the cell cycle progression in neoplastic tissues. Downregulation of HO-1 has been shown to inhibit the growth of a pancreatic tumor cell line [13], whereas overexpression of HO-1 in human and murine melanoma cells increased significantly their proliferation [20]. These effects seem to be strongly cell-type-dependent, as HO-1 activity exhibited antiproliferative actions in human and rat breast cancer cell lines [23]. HO-1 can also promote tumor angiogenesis and metastasis as demonstrated in humans [11,24] and in animal models [20,22,25]. Its overexpression in melanoma cells [20] and pancreatic cancer cells [26] increased colonization of lungs, whereas inhibition of HO activity completely inhibited formation of pulmonary metastases [26].

In contrast to the well-known role of HO-1 in growing tumors, data concerning its possible influence on tumor induction are very limited. Some indications come from clinical studies on polymorphisms of the HO-1 promoter, in which the presence of less active allelic variants seems to correlate with a higher incidence of lung cancer or squamous cell carcinoma (SCC) in smokers or areca chewers, respectively [27,28], suggesting a protective role for HO-1 in cancer induction. This supposition has not been verified under controlled, experimental conditions. Therefore, our aim was to examine the effect of HO-1 on the induction of squamous cell carcinoma in mice subjected to the two-step model of chemical carcinogenesis.

## Materials and methods

### Reagents

7,12-Dimethylbenz[*a*]anthracene (DMBA), phorbol 12-myristate 13-acetate (PMA), Triton X-100, phenylmethylsulfonyl fluoride, leupeptin, aprotinin, heparin, bicinchoninic acid protein assay kit, eosin, hematoxylin, and SYBR Green Jump Start Taq Ready Mix for High Throughput QPCR were purchased from Sigma (Poznan, Poland). Acetone, chloroform, ethanol, and xylene were procured from Polskie Odczynniki Chemiczne (Gliwice, Poland). Qiazol was obtained from Qiagen (Hilden, Germany) and BrdU Cell Proliferation ELISA from Roche Diagnostics (Warsaw, Poland). A total RNA extraction kit and reverse transcription system were obtained from Promega (Gdansk, Poland). ELISA kits for mouse vascular endothelial growth factor (VEGF), VEGF receptor-1 (VEGFR-1), tumor necrosis factor (TNF), sTNF-RI, keratinocyte attractant (KC), interleukin-1β (IL-1β), IL-6, IL-12, SDF-1α (stromal cell-derived factor-1α), and granulocyte colony stimulating factor (G-CSF) were procured from R&D Systems (Warsaw, Poland). Antibody for proliferating cell nuclear antigen (PCNA) detection was purchased from DAKO (Warsaw Poland) and FACS lysing solution from Becton–Dickinson (Gliwice, Poland).

### Animals

Experiments were performed on HO-1^−/−^ (*N* = 11), HO-1^+/−^ (*N* = 13), and HO-1^+/+^ (*N* = 12) females of C57BL/6 × FVB mice, generated from the HO-1^+/−^ breeding pairs kindly gifted by Dr. Anupam Agarwal (Birmingham, AL, USA). The first treatment was conducted on mice at the age of 9 weeks. All procedures were approved by the Institutional Animal Care and Use Committee at Jagiellonian University.

### Chemical carcinogenesis

A single dose of DMBA (100 nmol in 100 μl of acetone) was applied on shaved dorsal skin. Applications of PMA (5 nmol in 100 μl of acetone) started 2 weeks later and were repeated twice a week for 28 weeks. All manipulations were performed under anesthesia induced by inhaled isoflurane. Once a week the animals were weighed, and tumor sizes and locations were documented. Tumor volumes were determined by means of caliper and calculated using the following formula: *V* = *D* × *d*^2^ × 0.52 (*V* is the tumor volume, *D* is the biggest dimension; *d* is the smallest dimension).

### Hematoxylin–eosin (H&E) analysis

Skin tumors, collected at the end of the experiment (28th week) or on the day of death of particular animals (12–27 weeks), were fixed in neutral buffered formalin, embedded in paraffin, sectioned (6 μm), and stained with H&E. All slides were investigated in a blinded fashion by two pathologists (A.R. and A.P.) and assessed for tumor architecture, keratinocyte differentiation, cytologic atypia, and inflammation. Tumors were classified as follows: (i) typical papillomas—tumors showing papillomatous architecture and consisting of well-differentiated keratinocytes without cytologic atypia, with mitotic figures limited to the basal and lower epidermis; (ii) atypical papillomas—tumors with similar architecture, but comprising keratinocytes with cytologic atypia and augmented mitotic figures not limited to the lower epidermis; (iii) SCC in situ—tumors demonstrating keratinocyte cytologic atypia and mitotic figures involving the entire epidermis; and (iv) invasive SCC—tumors in which the clear penetration or disruption of the basement membrane by malignant keratinocytes was detected. Additionally, the semiquantitative assessment of inflammatory infiltrate, erythrocyte extravasation, and necrotic areas (0—no, 1—weak, 2—moderate, 3—strong reaction) was performed in a blinded fashion by two pathologists (A.R. and A.P.).

### PCNA staining

Staining was performed on paraffin sections after heat-induced epitope retrieval (0.01 M citric acid, pH 6.0, 95 °C, 3 × 4 min). Specimens were blocked (10% goat serum in TBS, room temperature, 60 min) and incubated with primary antibody (diluted 1:200 in TBS) overnight at 4 °C. After being washed in TBS, the specimens were incubated for 1 h at room temperature with secondary antibody (diluted 1:200 in TBS) conjugated with Alexa Fluor-546. Then the specimens were embedded in 4′,6-diamidino-2-phenylindole-containing medium. Negative control was prepared with the primary antibody omitted.

### Preparation of tissue lysates

Tumors and fragments of the liver isolated from each animal were homogenized using an automatic tissue lyser (Qiagen) in ice-cold PBS containing 1% Triton X-100 and protease inhibitors (1 μg/ml phenylmethylsulfonyl fluoride, 1 μg/ml leupeptin, and 1 μg/ml aprotinin). Then the samples were incubated for 30 min on ice and centrifuged (21,000*g,* 10 min, 4 °C). Clear supernatants were collected and total protein concentrations were determined by the bicinchoninic acid protein assay kit, according to the vendor's protocol.

### ELISA

Concentrations of proangiogenic and proinflammatory cytokines were measured in blood sera or tumor lysates using colorimetric sandwich ELISA according to the vendor's instructions.

### RNA isolation and real-time PCR

Fragments of liver isolated from each animal were homogenized in 1 ml of Qiazol and mixed with 300 μl of chloroform. The obtained lysates were vortexed, incubated on ice for 30 min, and centrifuged (30 min, 10,000*g,* 4 °C). Then, an upper aqueous phase was collected and subjected to ethanol precipitation. The RNA pellet was dissolved in nuclease-free water. Reverse transcription was performed on 1 μg of total RNA for 1 h at 42 °C using oligo(dT) primers and AMV reverse transcriptase. Real-time PCR was carried out using a Rotor Gene RG-3000 (Corbett Research) in a mixture containing SYBR Green PCR Master Mix, specific primers, and 30 ng of cDNA in a total volume of 15 μl. PCRs were performed in duplicate using a 5-min incubation at 94 °C followed by 40 three-step cycles of 30 s at 94 °C, 60 s at 60 °C, and 45 s at 72 °C. Relative gene expression was calculated with the Δ*C*_T_ method, with the elongation factor-2 (EF2) gene used as an internal control. The following primers were used: 5′-GACATCACCAAGGGTGTGCA (EF2 F), 5′-TCAGCACACTGGCATAGAGG (EF2 R); 5′-GTGGAGACGCTTTACGTAGTGC (HO-1 F), 5′-CTTTCAGAAGGGTCAGGTGTCC (HO-1 R); 5′-TGACCGAGCAGAAAATACCC (HO-2, F), 5′-GAAGTAAAGTGCAGTGGTGGC (HO-2, R); 5′-CCCCAAGATCCCGAACCTCT (biliverdin reductase, BVR F), 5′-TCAAGGCTCCCAAGTTCTCGTC (BVR R); 5′-GCTAGAGAAGATGGTCGCCAAGCAG (thioredoxin-2, Thrx-2 F), 5′-TCCTCGTCCTTGATCCCCACAAACTTG (Thrx-2 R); 5′-TTTTTGCGCGGTCCTTTCC (superoxide dysmutase-1, SOD-1 F), 5′-ATGGACGTGGAACCCATGCT (SOD-1 R); 5′-AATCTCAACGCCACCGAGGA (SOD-2 F), 5′-TCTCCTTTGGGTTCTCCACCA (SOD-2 R); 5′-CTTCGAGCCTGAGCCCTTTG (ferritin F), 5′-CAGGTTGATCTGGCGGTTGA (ferritin R); 5′-TGACATGGTCTGGGACTTCTGG (catalase F), 5′-TTGATGCCCTGGTCGGTCTT (catalase R); 5′-CCCCTTTCCCTCTGCTGAAG (glutathione *S*-transferase-1, GSTA-1 F), 5′-TGCAGCTTCACTGAATCTTGAAAGC (GSTA-1 R); 5′-GGCACTTGCGTGAATGTTGG (glutathione reductase, GR F), 5′-GGCATCCCTTTTCTGCTTGATG (GR R); 5′-GTCCAATCCTGGTGATGT (p21 F), 5′-GTTTTCGGCCCTGAGATG (p21 R); 5′-ACTACCTGGACCGCTTCCTGT (cyclin D1 F), 5′-GATGAAATCGTGGGGAGTCATG (cyclin D1 R); 5′-ATTTCAAGTGCGTGCAGAAGGA (cyclin D2, F), 5′-GTCAGCGGGATGGTCTCTTT (cyclin D2, R); 5′-CTCCTACTTCCAGTGCGTCAA (cyclin D3, F), 5′-CAATAGTCAGGGGCGTGGTTTC (cyclin D3 R).

### Gene profile screening

An expression profile of genes regulating the cell cycle was analyzed using the GEArray Q series assay (SuperArray Biosciences, Bethesda, MD, USA) according to the vendor's protocol.

### Isolation of primary fibroblasts

Analysis of gene expression in tumors is hampered by heterogeneity of the tissue. Therefore, to investigate the influence of HO-1 on the cellular response to DMBA, we isolated primary fibroblasts from the skin of mice and incubated them in the presence of DMBA (5 μM). Additionally, to investigate the role of HO-1 in cell transformation, we transduced the fibroblasts with c-myc, using retroviral vectors.

Fragments of skin taken from murine newborns were digested for 6–8 h at 37 °C with a mixture containing collagenase type II (1.5 mg/ml) dissolved in Dulbecco's modified Eagle's medium (DMEM) LG supplemented with penicillin (100 U/ml), streptomycin (100 μg/ml), Glutamax (2 mM), and 20% fetal bovine serum (FBS; for later passages and regular culture 10% FBS was applied). The obtained cell suspension was gently mixed and centrifuged (1100*g,* 10 min, at room temperature). Supernatant was discarded, whereas cells were resuspended in culture medium and plated. On the third day fibroblasts were washed with PBS and cultured under standard conditions. Cells at passages 3 and 4 were used for experiments.

### Production of retroviral vectors and transduction of fibroblasts

Retroviral vectors were produced using the Phoenix-Eco HEK293 packaging cell line. Cells were cotransfected with plasmids pBabe encoding the murine c-myc gene and pM13 harboring the gag and pol genes, using SuperFect, according to the vendor's protocol. Control vectors were produced using pM13 and pLZRS plasmids bearing the enhanced yellow fluorescent protein (EYFP) gene. Media containing retroviruses were collected 48 h after cotransfection and frozen at − 80 °C or immediately mixed with an equal volume of DMEM supplemented with Polybrene (4 μg/ml) and used for transduction of fibroblasts.

Primary fibroblasts were grown to ~ 60% confluency. Just before transduction the cells were washed with PBS and overlaid with retroviral solution for 3 days. Afterward viruses were removed, and the fibroblasts were cultured for the next 3 days and then subjected to RNA or protein isolation. In each experiment the efficacy of transduction was ~ 30% for all genotypes as assessed in control cells treated with retroviral vectors harboring the EYFP reporter gene.

### Kinase activity assay

Measurement of phosphorylation of Akt and S6 ribosomal protein was done using the PathScan Multiplex Western Cocktail I Detection Kit (Cell Signaling Technology, Warsaw, Poland) according to the manufacturer's instructions. In short: untreated or c-myc-overexpressing cells were seeded in six-well plates and grown to about 50% confluency. Then some samples were stimulated with DMBA at the final concentration of 5 μmol/L. Proteins were isolated after a 72-h incubation period.

Protein samples (15 μg) were diluted in dH_2_O to a final volume of 33 μl, mixed with 7 μl of Protein Loading Buffer, and denatured for 5 min at 95 °C. Then the proteins were separated by SDS–PAGE using 4% stacking and 12% running gels, at 100 and 200 V, respectively. Transfer to the nitrocellulose membrane was run at 4 °C for 1.5–2 h at 100 V. The membrane was stained with Ponceau S for 2 min to visualize the protein bands. Then, the stain was removed by triple washing with dH_2_O. The membrane was blocked in 5% nonfat dry milk in 1× TBS with 0.05% Tween 20 for 1 h at room temperature, washed six times for 5 min in 1× TBS with 0.05% Tween 20, and incubated overnight with primary rabbit antibody cocktail diluted 1:200 in a dilution buffer (5% albumin in 1× TBS with 0.05% Tween 20) at 4 °C. Next, the membrane was washed and incubated for 1 h with horseradish peroxidase (HRP)-conjugated secondary antibody (1:1000) in a dilution buffer (5% nonfat dry milk in 1× TBS with 0.05% Tween 20) at room temperature. Then, the membrane was washed and incubated with an HRP substrate for 5 min, followed by membrane development.

### Cell proliferation assay

Proliferation of fibroblasts was measured using a bromodeoxyuridine (BrdU) ELISA according to the vendor's protocol. Briefly: primary fibroblasts (5000 cells/well) were seeded in 96-well plates, starved for 24 h in the absence of FBS, and then stimulated with medium containing 10% FBS for the next 24 h. Unstimulated cells served as a controls. BrdU (final concentration of 10 μmol/L) was added to the cell cultures for the last 2 h. The medium was removed, and the cells were dried, fixed with FixDenat (100 μl per well, 30 min), and incubated with anti-BrdU antibody (1:100, 1.5 h, room temperature). The cells were washed three times and incubated with substrate solution (15 min, incubation at room temperature in darkness). The reaction was stopped with 1 M H_2_SO_4_ and absorbance was measured at the wavelength 450 nm.

### Cell motility assay

Migration of fibroblasts was analyzed using a modified Boyden chamber (8-μm pore diameter). Cells (150,000 per well) were seeded on the transwell insert in 300 μl of medium devoid of FBS but supplemented with 5% bovine serum albumin. To the lower part, 500 μl of the same medium with or without FBS (10%) was added. Migration was assessed 24 h later: cells on the lower surface of inserts were fixed and stained with crystal violet (CV) solution. Quantification of migration was performed by measurement of absorbance after methanol extraction of CV at the wavelength 570 nm.

### Semiliquid agarose clonogenic test

Agarose (0.8 and 0.4%) was dissolved in sterile water and autoclaved. Warmed 0.8% agarose was mixed with 2× DMEM (1:1 v/v) and 2 ml of the mixture was placed in each well of a six-well plate. Cell suspensions (2 × 10^4^ cells/ml) were prepared in 2× DMEM, mixed with 0.4% agarose (1:1 v/v), and gently poured on a 0.8% agarose layer (1 ml/well). Plates were incubated under standard conditions for 7 days and then colonies were counted under the microscope in 50 random fields of view. In all experiments, B16(F10) murine melanoma cells were used as a positive control, and untreated primary fibroblasts served as a negative control.

### Measurements of carbon monoxide

Tumor cells were seeded on plates (100,000 per well) and cultured for 48 h. Then, the cells were washed twice with PBS, scraped, and centrifuged (10,000*g,* 10 min, 4 °C). Pellets of cells were snap-frozen in liquid nitrogen and production of CO was quantified using gas chromatography as described elsewhere [29].

### Statistical analysis

Results are presented as means ± SEM of at least three independent experiments. Statistical significance was determined using Student's *t* test or Fisher's exact test. Kaplan–Meier analysis was used to compare the time course of observed effects.

## Results

### Effect of HO-1 expression on sensitivity to carcinogens

**Fig. 1:**
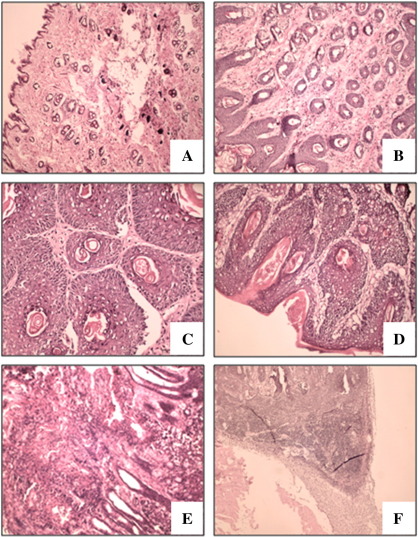
Histological analysis of tumors. Lesions were classified as follows: (A) normal epithelium, (B) hyperplasia, (C) papilloma, (D) atypicum papilloma, (E) carcinoma, and (F) metastases. Hematoxylin and eosin staining on paraffin-embedded sections. Original magnification: 170× (A), 340× (B, D, E), or 80× (F).

Treatment of mice with DMBA and PMA led to the development of skin lesions resembling papilloma, which gradually progressed to squamous cell carcinoma. This was confirmed by macroscopic observations and by morphological analysis of paraffin-embedded tissues stained with H&E, by which specimens were classified as normal epithelium (Fig. 1A), hyperplasia (Fig. 1B), papilloma (Fig. 1C), papilloma atypicum (Fig. 1D), or carcinoma (Fig. 1E). Metastatic infiltration to the local lymph nodes was observed in mice with the most advanced tumors (Fig. 1F).

**Fig. 2:**
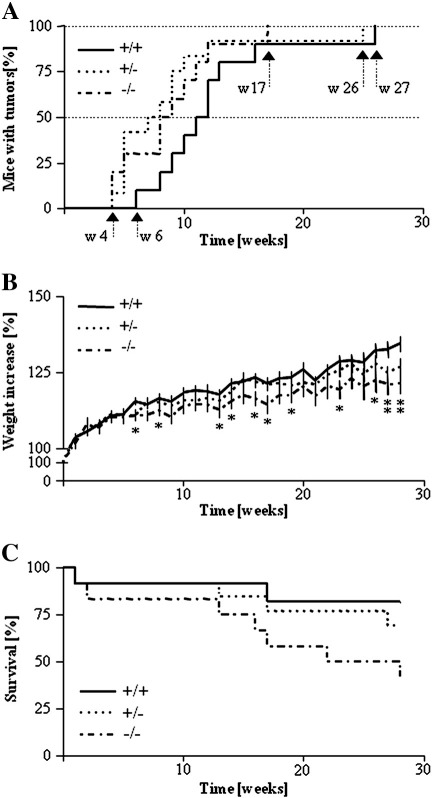
Tumor development and survival of mice of different HO-1 genotypes treated with DMBA/PMA. (A) Time course of latency to the first tumor (*P* < 0.05 HO-1^+/−^ vs HO-1^+/+^, *P* = 0.061 HO-1^−/−^ vs HO-1^+/+^). Each point represents the mean ± SEM. (B) Time course of body weight increase (*P < 0.05 HO-1^−/−^ vs HO-1^+/+^). (C) Survival of mice (*P* < 0.05 HO-1^−/−^ vs HO-1^+/+^).

Experiments were performed in mice of different HO-1 genotypes. Importantly, the first lesions appeared 2 weeks later in the wild-type group than in their HO-1^−/−^ and HO-1^+/−^ counterparts (Fig. 2A). Such delay in induction of papilloma development was observed in animals with a normal level of HO-1 during the whole time course of the experiment (*P* < 0.05 for comparison of HO-1^+/+^ and HO-1^+/−^ and *P* = 0.061 for comparison of HO-1^+/+^ and HO-1^−/−^, Kaplan–Meier test). Thus, induction of lesions in 50% of the treated animals required recurrent PMA applications for 9 weeks in the HO-1^−/−^, 8 in the HO-1^+/−^, and 12 in the HO-1^+/+^ mice, whereas tumors appeared in all individuals respectively after 17, 25, and 26 weeks. We also found that lack of HO-1 expression was associated with reduced weight gain (Fig. 2B) and increased mortality of animals (Fig. 2C). Thus, survival rate at the end of the experiment was 82% in HO-1^+/+^, 69.2% in HO-1^+/−^, and only 41.7% in HO-1^−/−^ individuals (*P* < 0.05 for comparison of HO-1^+/+^ and HO-1^−/−^, Kaplan–Meier test).

**Fig. 3:**
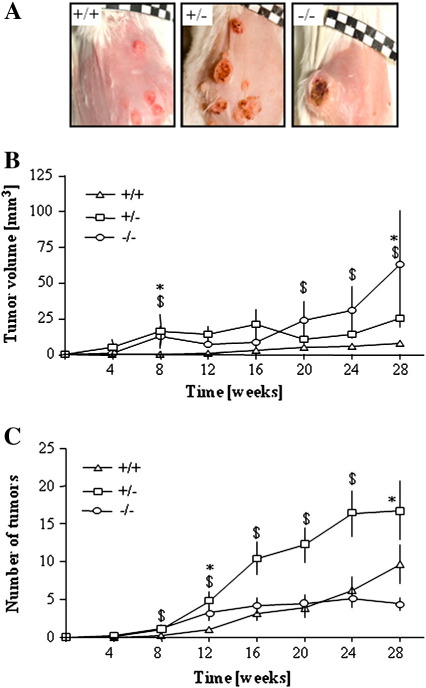
Sizes and numbers of DMBA/PMA-induced tumors in mice of various HO-1 genotypes. (A) Representative photos of tumors at the end of the experiment. (B) Mean volume of tumors. (C) Mean number of tumors (*P < 0.05 HO-1^−/−^ vs HO-1^+/+^, $*P* < 0.05 HO-1^+/−^ vs HO-1^+/+^). Each point represents the mean ± SE.

The volume of nodules in the wild-type animals increased much more slowly than in the other genotypes, and even at the last week of experiments the tumors remained small (Figs. 3A and B). Interestingly, there were apparent differences in lesion progression between HO-1^+/−^ and HO-1^−/−^ mice. Low levels of HO-1 expression in HO-1^+/−^ animals resulted in the generation of more numerous but still relatively small nodules (Figs. 3A, B, and C). A complete lack of HO-1 in HO-1^−/−^ individuals led to the formation of few (sometimes single) tumors, but their volume was large, much bigger than in the other experimental groups (Figs. 3A, B, and C). At the 28th week of treatment the average tumor volume was 8.62 ± 1.91 mm^3^ in HO-1^+/+^, 25.52 ± 9.38 mm^3^ in HO-1^+/−^ (*P* < 0.01, Student's *t* test), and 63.12 ± 42.55 mm^3^ in HO-1^−/−^ mice (*P* < 0.05, Student's *t* test). Taken together, these observations indicate that expression of HO-1 may protect animals from the DMBA/PMA-induced skin injury and development of lesions.

It should be kept in mind, however, that HO-1 is not the only source of HO activity. Measurement of CO production in cells isolated from the tumors and cultured in vitro showed only an ~ 45% decrease in CO generation by HO-1^−/−^ cells (10.2 and 5.7 nmol/h/mg protein in HO-1^+/+^ and HO-1^−/−^ cells, respectively). This indicates relatively high activity of HO-2. Accordingly, qRT-PCR analysis confirmed the expression of HO-2 in tumors, at similar levels in HO-1^+/+^ and HO-1^−/−^ samples (respectively 0.259 ± 0.055 and 0.207 ± 0.044, HO-2/EF2 relative expression).

Surprisingly, pathomorphological analysis of H&E-stained specimens evidenced that the nodules on HO-1^−/−^ mice, although large, displayed features of benign papilloma. Namely, at the end of the experiment, dysplastic foci in tumors were observed in only 1 of 6 HO-1^−/−^ individuals (16.7%). In contrast, even low levels of HO-1 in lesions (in HO-1^+/−^ animals) were associated with a higher grade of tumor malignancy, suggesting that expression of HO-1 may facilitate malignant transformation of growing tumors. Dysplastic foci or features typical of infiltrating squamous cell carcinoma were found in 8 of 9 HO-1^+/−^ individuals (88.9%) and in all 10 HO-1^+/+^ mice. Differences between genotypes were statistically significant (*P* < 0.001, Fisher's exact test).

### Effect of HO-1 on expression of inflammatory and angiogenic factors

**Fig. 4:**
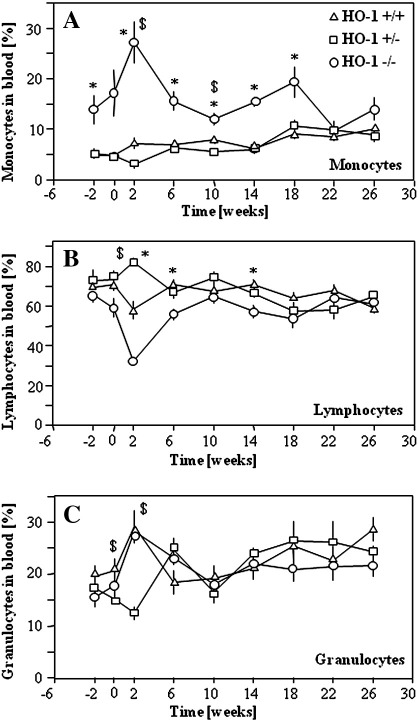
Percentages of (A) monocyte, (B) lymphocyte, and (C) granulocyte subpopulations in the blood of mice of various HO-1 genotypes treated with DMBA/PMA. FACS analysis of SSC/FSC dot-blots (*P < 0.05 HO-1^−/−^ vs HO-1^+/+^, $*P* < 0.05 HO-1^+/−^ vs HO-1^+/+^). Each point represents the mean ± SE.

To monitor the carcinogen-induced systemic inflammatory reaction of mice we periodically checked the frequency of major leukocyte populations in the peripheral blood, using flow cytometric SSC/FSC dot-plot analysis (Figs. 4A–C). In healthy HO-1^−/−^ animals the frequency of monocytes was higher than in the two other groups (Fig. 4A). Similarly, 2 weeks after topical application of DMBA monocytes constituted 4.7 ± 0.6, 4.7 ± 0.6, and 17.1 ± 5.2% of peripheral blood leukocytes in HO-1^+/+^, HO-1^+/−^, and HO-1^−/−^ mice. Two weeks after the first PMA treatment the level was raised to 27.2 ± 5.9% in HO-1 knockouts, whereas it remained lower in heterozygous (7.3 ± 1.1%) and wild-type mice (3.1 ± 0.4%). Starting from the 10th week of treatment the percentage of monocytes in HO-1-deficient mice returned to the initial value. Then it gradually rose until week 18, but again decreased at the end of the experiment (Fig. 4A). There were no differences in the frequency of leukocyte subpopulations between animals that survived until the 28th week or died in the course of the experiment.

**Fig. 5:**
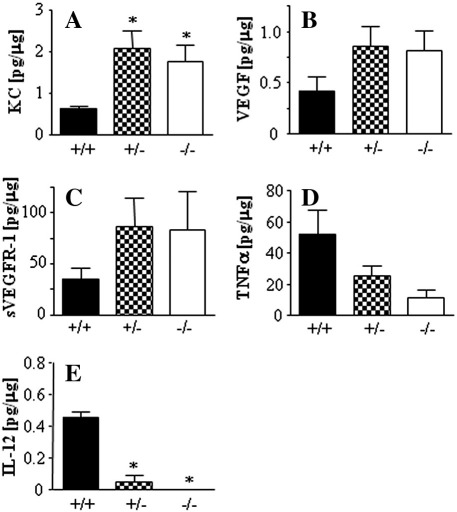
Concentrations of (A) KC, (B) VEGF, (C) VEGF-R1, (D) TNFα, and (E) IL-12 in tumor lysates measured using colorimetric ELISA at the end of the experiment (*P < 0.05 vs HO-1^+/+^). Each bar represents the mean + SE.

We also measured the local concentrations of proinflammatory and proangiogenic cytokines in tumors. Thus, the nodules growing in wild-type animals tended to produce less KC, VEGF, and VEGF-R1 (Figs. 5A, B, and C) than HO-1^+/−^ or HO-1^−/−^ tumors. In contrast, they contained higher levels of TNFα and IL-12, proteins displaying some antitumoral activities (Figs. 5D and E). Production of IL-6, SDF-1α, and G-CSF was similar in tumors of the various HO-1 genotypes (data not shown).

Despite the distinct production of proinflammatory cytokines, pathomorphological investigation did not show any apparent differences in the inflammatory infiltrates. The semiquantitative assessment showed that the average grade of inflammatory infiltration was 1.250 ± 0.229, 1.0 ± 0.365, and 1.670 ± 0.333 in HO-1^+/+^, HO-1^+/−^, and HO-1^−/−^ individuals, respectively. The infiltrates contained mononuclear cells, neutrophils, eosinophils, and mast cells and were similar in tumors of all genotypes.

**Fig. S1:**
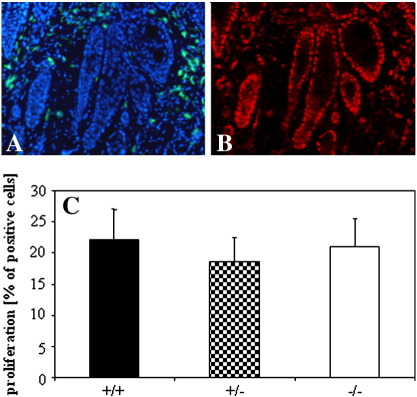
Proliferation of tumor cells and vascularization of tumors. A – DAPI (blue – nuclei) and isolectin (green – endothelial cells) staining, B – PCNA (red – proliferating cells) staining, C – calculation of proliferation rate in HO-1^+/+^, HO-1^+/−^, and HO-1^−/−^ tumors. Each bar represents mean + SE.

Labeling of endothelial cells using isolectin binding has demonstrated that all lesions were well vascularized (Supplementary Fig. S1A). The results did not show, however, any genome-specific differences. Also in pathomorphological assessments no clear differences in vascularization were noticed (data not shown). Nevertheless, semiquantitative evaluation of erythrocyte extravasation suggested an HO-1-dependent trend, in which HO-1-deficient mice had the lower rate of intratumoral bleeding (the average extravasation grade was 0.615 ± 0.320, 0.250 ± 0.207, and 0.200 ± 0.211 for HO-1^+/+^, HO-1^+/−^, and HO-1^−/−^ animals, respectively).

Unexpectedly, although HO-1-deficient tumors grew faster, we were unable to find any differences in the number of proliferating, PCNA-positive cells in HO-1^+/+^, HO-1^+/−^, and HO-1^−/−^ mice (Supplementary Fig. S1). Also analysis of mitogenic figures in histological specimens did not indicate the genotype-related trends (data not shown).

Additionally we performed semiquantitative histological analysis of necrotic areas. Here we found a trend toward the higher necrosis rate in HO-1-deficient animals: the average necrosis grade was 0 ± 0, 0.167 ± 0.192, and 0.400 ± 0.281 for HO-1^+/+^, HO-1^+/−^, and HO-1^−/−^ mice, respectively. It is difficult to judge, however, if the more pronounced necrosis in HO-1^−/−^ tumors was a direct consequence of HO-1 deficiency and increased cell mortality or an indirect effect of larger volumes of tumors.

### Effect of DMBA/PMA treatment on expression of cytoprotective genes

**Fig. 6:**
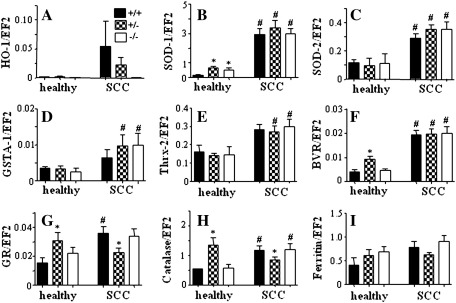
Expression of cytoprotective genes in the livers of healthy and DMBA/PMA-treated mice of various genotypes measured using qRT-PCR. (A) HO-1, (B) SOD-1, (C) SOD-2, (D) GSTA-1, (E) Thrx-2, (F) BVR, (G) GR, (H) catalase, (I) ferritin. EF2 was used as a constitutive housekeeping gene (*P < 0.05 vs HO-1^+/+^, #*P* < 0.05 vs healthy mice). Each bar represents the mean + SE.

Because local application of DMBA/PMA can potentially induce a systemic oxidative stress, and HO-1-deficient mice are more prone to systemic oxidative challenge, we checked the expression of antioxidative genes in the livers. Using qRT-PCR we compared the expression patterns in healthy animals and in mice after the 28-week exposure to carcinogens. First, we found that the level of HO-1 in healthy individuals was very low and similar in HO-1^+/+^ and HO-1^+/−^ mice. In response to carcinogens it increased potently, with the strongest reaction in wild-type and much weaker in heterozygous animals (Fig. 6A). Carcinogens led also to significant upregulation of SOD-1, SOD-2, GSTA-1, Thrx-2, and BVR, to similar extents in mice of all HO-1 genotypes (Figs. 6B–F). On the other hand, expression of GR and catalase was higher in the healthy HO-1^+/−^ group than in HO-1^+/+^ and HO-1^−/−^ (Figs. 6G and H). In response to carcinogens it was significantly augmented in the wild-type and knockout mice, but remained relatively stable in heterozygotes. Ferritin was similarly expressed before and after treatment in all experimental groups (Fig. 6I). These changes may indicate a systemic oxidative stress induced by carcinogens, which is not meaningfully influenced by the level of HO-1 expression. It is also suggested that the differences observed in the development of tumors do not result from different levels of systemic oxidative response in HO-1^+/+^, HO-1^+/−^, and HO-1^−/−^ animals.

### Effect of DMBA on cultured fibroblasts

**Fig. S2:**
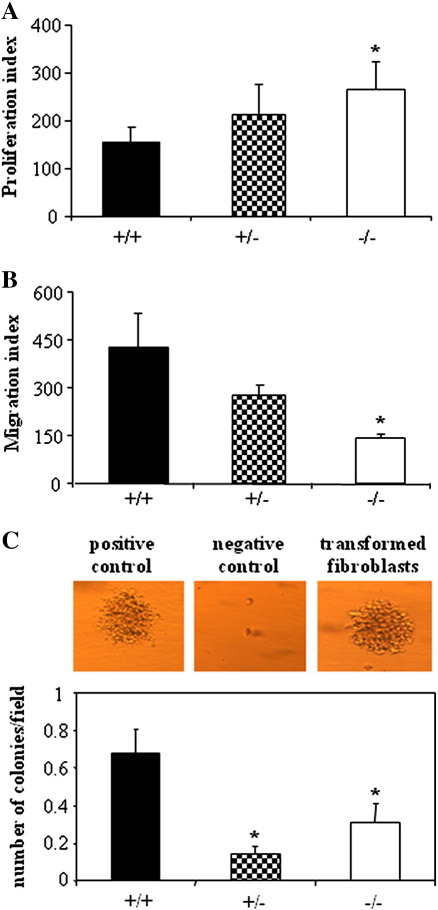
Activities of primary fibroblasts isolated from mice of different genotype and cultured *in vitro*. A – Proliferation of fibroblasts measured using BrdU ELISA. Cells were starved for 24 h in empty medium and then stimulated for 24 with 10% FCS. B – FCS-induced migration of fibroblasts analyzed using modified Boyden chambers. C – Efficacy of transformation of fibroblasts after retroviral c-myc overexpression measured using clonogenic. Cells were cultured in semiliquid agarose for two weeks. Pictures show positive control used in the assay (B16(F10) melanoma cells), negative control (non transformed fibroblasts) and typical colony formed by the transformed fibraoblasts (* – P < 0.05 *vs.* HO-1^+/+^). Each bar represents mean + SE.

Neither incubation with DMBA for 24–72 h nor treatment with c-Myc influenced significantly viability or proliferation of primary skin fibroblasts of the various genotypes, as estimated by 3-(4,5-dimethylthiazol-2-yl)-2,5-diphenyltetrazolium bromide reduction, lactate dehydrogenase activity, and BrdU incorporation assays (data not shown). Interestingly, in vitro experiments showed that HO-1 deficiency may increase the FBS-induced proliferation of fibroblasts (Supplementary Fig. S2A) and reduced their motility (Supplementary Fig. S2B). Interestingly, HO-1 expression strongly influenced the ability of c-myc-transformed fibroblasts to grow in semiliquid agarose in clonogenic assays. Wild-type cells were able to form many more colonies than HO-1^+/−^ or HO-1^−/−^ cells (Supplementary Fig. S2C). Treatment with DMBA, regardless of concentration and time of exposure, did not transform the fibroblasts in vitro (data not shown).

Neither incubation with DMBA for 24–72 h nor treatment with c-Myc influenced significantly viability or proliferation of primary skin fibroblasts of the various genotypes, as estimated by 3-(4,5-dimethylthiazol-2-yl)-2,5-diphenyltetrazolium bromide reduction, lactate dehydrogenase activity, and BrdU incorporation assays (data not shown). Interestingly, in vitro experiments showed that HO-1 deficiency may increase the FBS-induced proliferation of fibroblasts (Supplementary Fig. S2A) and reduced their motility (Supplementary Fig. S2B). Interestingly, HO-1 expression strongly influenced the ability of c-myc-transformed fibroblasts to grow in semiliquid agarose in clonogenic assays. Wild-type cells were able to form many more colonies than HO-1^+/−^ or HO-1^−/−^ cells (Supplementary Fig. S2C). Treatment with DMBA, regardless of concentration and time of exposure, did not transform the fibroblasts in vitro (data not shown).

**Fig. S3:**
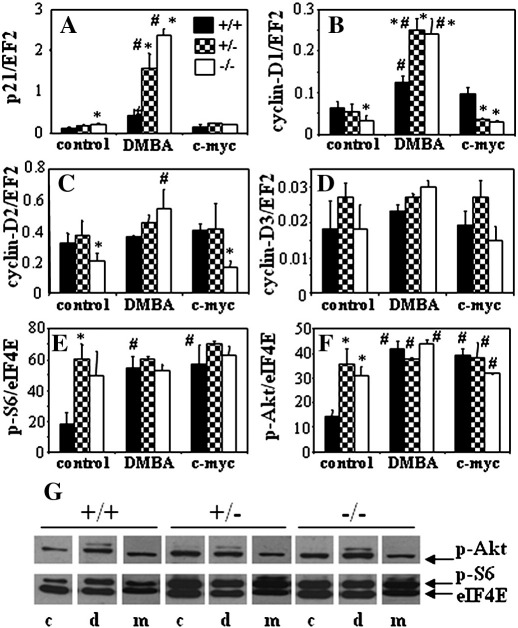
Expressions of genes (measured using qRT-PCR) and activities of kinases (assessed by densytometric analysis of western-blots) involved in cell-cycle regulation in fibroblasts isolated from mice of different HO-1 genotype and cultured *in vitro*. Cells were stimulated with DMBA (5 μM) for 72 h or transduced with retroviral vectors harboring c-myc and then cultured for 72 h. A – p21, B – cyclin-D1, C – cyclin-D2, D – cyclin-D3, E – phosphorylated Akt, F – phosphorylated S6. G – representative blots showing p-Akt and p-S6: c – control; d – cells treated with DMBA; m – cells overexpressing c-myc. EF2 and eIF4E were used as a constitutive housekeeping genes and loading controls in qTY-PCR and western-blotting, respectively (* – P < 0.05 *vs.* HO-1^+/+^, # - P < 0.05 *vs.* control, unstimulated). Each bar represents mean + SE.

Then, we used the GEArray Q series assay (SuperArray Biosciences) to screen the expression profile of 23 genes regulating cell cycle in the primary fibroblasts. This allowed us to select the p21 and cyclin-D genes, which were differentially expressed in HO-1^+/+^, HO-1^+/−^, and HO-1^−/−^ cells. Quantitative RT-PCR analysis confirmed that lack of HO-1 in resting cells was associated with increased expression of p21 and reduced expression of cyclin-D1 and cyclin-D2. The level of cyclin-D3 turned out to be independent of HO-1 status (Supplementary Figs. S3A–D). DMBA (5 μM, 72 h) only weakly upregulated p21 expression in the wild-type cells, but strongly increased it in the HO-1^+/−^ and HO-1^−/−^ fibroblasts. Transformation with c-myc did not influence p21 in any group (Supplementary Fig. S3A).

Cyclin-D1 was upregulated by DMBA in a similar way—the effect was much stronger in the HO-1^+/−^ and HO-1^−/−^ than in the wild-type cells. Interestingly, expression of cyclin-D1 after c-myc transformation was higher in HO-1^+/+^ than in HO-1^+/−^ and HO-1^−/−^ fibroblasts (Supplementary Fig. S3B). A similar tendency was found for cyclin-D2—the upregulation in response to DMBA was apparent only in HO-1^−/−^ fibroblasts, whereas in cells transduced with c-myc, like in the control cells, cyclin-D2 remained at a much lower level (Supplementary Fig. S3C). Expression of cyclin-D3 was not significantly influenced by DMBA or c-Myc, regardless of the HO-1 status (Supplementary Fig. S3D). We also demonstrated that exposure of the wild-type fibroblasts to DMBA or their transduction with c-myc strongly activates Akt and S6 kinases. In contrast, either HO-1^+/−^ or HO-1^−/−^ cells displayed much higher activation of both kinases under control conditions, which was not further augmented in response to the treatments (Supplementary Figs. S3E, F, and G).

## Discussion

The major findings of our study are: (i) HO-1-deficient mice are more vulnerable to DMBA/PMA-induced skin injury, (ii) lack of HO-1 results in the development of large, but benign, papillomas in response to DMBA/PMA treatment, whereas HO-1 expression seems to facilitate a transformation of growing tumors to malignant carcinoma; and (iii) HO-1-deficient fibroblasts exposed to DMBA demonstrate more pronounced upregulation of p21, cyclin-D1, and cyclin-D2 expression, but are less susceptible to c-myc-mediated transformation. Thus, HO-1 can protect healthy skin from the initial injury caused by carcinogens, but in growing tumors it can promote malignancy.

An association between induction of HO-1 and tumor growth or malignancy was indicated already in 1997 by Goodman and colleagues [7], who demonstrated a severalfold elevation of HO-1 expression in rapidly growing renal carcinoma cells. This increase could be induced by a factor(s) released by the tumors or could be a general response to oxidative stress or hypoxia. Generally, it appeared to promote tumor progression [7].

We demonstrated that mice with a low or no HO-1 expression (HO-1^+/−^ and HO-1^−/−^) developed the first DMBA/PMA-induced lesions earlier then their wild-type counterparts. Indications of a similar, potentially protective role in chemically induced carcinogenesis were reported for other antioxidative genes, NADPH:quinone oxidoreductase-1 (NQO-1) and NF-E2-related factor (Nrf2) [30,31]. Namely, treatment with DMBA alone led to the development of skin tumors in about 50% of the NQO-1^−/−^ mice but in none of the wild-type individuals [30]. Also, when the DMBA/PMA two-step model of carcinogenesis was used, the NQO-1^−/−^ mice developed larger tumors at a greater frequency than their wild-type littermates [31]. Likewise, intragastric administration of benzo[*a*]pyrene to Nrf2-deficient mice resulted in a significantly higher burden of gastric neoplasia than in wild-type animals [31].

Our results also support the clinical analyses, in which carriers of more active variants of the HO-1 promoter were more frequent among healthy subjects than among patients suffering from oral squamous cell carcinoma in betel chewers, lung adenocarcinoma in heavy smokers, and breast carcinoma in women subjected to iron supplementation [27,28,32–35]. This suggests a protective role for HO-1 at least against carcinogens associated with induction of oxidative stress.

Although mean latency to the first lesion is very similar in HO-1^−/−^ and HO-1^+/−^ animals, the resulting tumors are different: HO-1^−/−^ mice develop a few, but large, lesions, and HO-1^+/−^ mice much smaller but very numerous ones. The mechanism underlying these differences is not clear. One can hypothesize that lack of HO-1 may lead to a higher apoptotic rate of affected cells in skin treated with carcinogens and thereby to a reduced rate of initiation, whereas after transformation it may facilitate cell proliferation. This supposition might be supported by the observation of increased proliferation of fibroblasts isolated from HO-1^−/−^ mice, although it should be kept in mind that the influence of HO-1 on the cell cycle is tissue-specific [32]. Additionally, we noticed the augmented activity of Akt kinase and its effector S6 ribosomal protein in resting HO-1^+/−^ and HO-1^−/−^ cells. This pathway regulates proliferation, apoptosis, and growth in a variety of cell types [36]. Its inhibition contributes to the antineoplastic effects of several drugs, such as dibenzoylmethane [37], enzastaurin [38], resveratrol [39], xanthorrhizol [40], or bromelain [41]. Expression of constitutively active Akt increases the susceptibility of mice to the induction of mammary tumors of epithelial origin by DMBA [36]. Thus it seems possible that the increased activity of Akt in HO-1-deficient cells might facilitate the growth of tumors.

HO-1 knockout mice displayed the highest mortality and the smallest body weight gain in the time course of the experiments. However, large tumors developed by HO-1^−/−^ individuals remained benign papillomas, in contrast to the dysplastic lesions found in HO-1^+/−^ and HO-1^+/+^ mice. It must be stressed that the low number of HO-1^−/−^ animals with malignant carcinoma at the end of the experiment did not result from an earlier death of individuals with advanced tumors in this group. Apart from tumors harvested from the surviving animals at the end of the study (at week 28), we also collected tumors from animals that died earlier, starting from week 13. The results (not shown) were the same as for tumors taken on week 28: all HO-1^−/−^ animals dying between weeks 13 and 28 developed benign lesions, whereas the first carcinoma in a wild-type individual was found already at week 16. Therefore, the differences between the proportions of malignant tumors in mice of different genotypes were not caused by preterm deaths of HO-1^−/−^ animals with more advanced tumors and survival of those with milder lesions.

A discrepancy between the growth and the malignancy of tumors was also observed in p53-deficient mice. In this model, opposite to HO-1 deficiency, the p53 knockout mice developed smaller DMBA-induced papillomas than their wild-type counterparts. However, the malignant conversion of these small papillomas to squamous cell carcinomas was markedly accelerated [42].

We suppose that increased mortality of HO-1 knockouts observed in the last part of the experiment might not be directly associated with tumor malignancy, but rather might be caused by a higher sensitivity to inflammatory response to PMA treatment, especially in older animals. Indeed, Poss and Tonegawa have shown that HO-1-deficient mice develop chronic inflammation and augmented sensitivity to oxidative injury, the effect increasing with age [43]. Here we demonstrated that HO-1 deficiency was also associated with a higher proportion of monocytes in the blood of untreated animals and with a much stronger increase in monocyte fraction in response to the first PMA treatment, which may indicate a stronger inflammatory response.

We also found that the local concentrations of VEGF were higher in HO-1-deficient than in wild-type individuals. This was surprising, because VEGF is positively regulated by HO-1 in many cell types cultured in vitro [3], and its upregulation can mediate the proangiogenic activities of HO-1 in some cancers [11,20,24,26]. Possibly, the higher tumoral concentration of VEGF in the HO-1-deficient mice is rather an indirect effect, resulting, for example, from upregulation of some proinflammatory mediators. In contrast to the healthy tissues, in the inflamed organs HO-1 is known to inhibit VEGF production [44]. One such mediator might be KC, the murine ortholog of human IL-8, which directly upregulates VEGF expression in an NF-κB-dependent manner [45]. The KC concentration was much higher in HO-1^−/−^ and HO-1^+/−^ than in HO-1^+/+^ mice (Fig. 5A). Alternatively, larger tumors growing in HO-1-deficient mice could be more hypoxic and therefore might produce more VEGF. This supposition might be supported by a very similar increase in expression of VEGF-R1 (Fig. 5C), which is directly upregulated by hypoxia [46,47].

It seems that production of proinflammatory cytokines is not a significant factor influencing the malignant conversion of papilloma in our model. In HO-1^+/−^ and HO-1^−/−^ tumors, the local concentrations of all cytokines studied were similar, whereas the frequency of dysplastic foci was much higher in heterozygotes. It has been reported that conversion of papilloma to SCC can be accelerated by oxidative stress, as antioxidants inhibit PMA-dependent promotion of carcinogenesis [48–49]. We observed an upregulation of several antioxidative enzymes in the livers of animals treated with DMBA/PMA, which indicates the induction of systemic oxidative stress. However this response was similar in all experimental groups, regardless of the HO-1 genotype. Thus, it seems that the antioxidative potential of HO-1 does not play a major role in our system. Furthermore, a lack of HO-1 did not facilitate but actually attenuated the malignant conversion.

Treatment with DMBA is known to induce p21 and cyclin-D1 in carcinoma [50]. Experiments performed on primary fibroblasts isolated from the skin of mice of the various HO-1 genotypes confirmed the induction of p21 and cyclin-D1 and demonstrated that this response was much stronger in HO-1^+/−^ and especially in HO-1^−/−^ cells than in their wild-type counterparts. Thus, it seems that HO-1 may to some extent attenuate the effects of DMBA, which we also observed in the growing tumors in vivo. Interestingly, it was reported that p21 deficiency in DMBA-treated mice resulted in the development of more undifferentiated tumors, with a high frequency of anaplastic spindle cell carcinomas, indicating the protective role of p21 against progressive malignancy [51]. We found the lowest rate of dysplastic conversion in HO-1 knockouts, in which the fibroblastic expression of p21 was the highest, which might suggest the involvement of this pathway in HO-1-dependent differences in DMBA/PMA carcinogenesis. However, in contrast to in vitro cell cultures, we did not find statistically significant differences in p21 expression in heterogeneous tumor tissue (data not shown).

We also compared the influence of HO-1 on the transformation of primary fibroblasts overexpressing c-myc after retroviral transduction. This proto-oncogene is a key player in the cell cycle, acting among others through inhibition of p21 and upregulation of cyclins-D [52–54]. In contrast to modulation of DMBA activity, lack of HO-1 reduced the effect of c-myc overexpression on the cell-cycle-regulating genes. Thus, the levels of cyclin-D1 and -D2 were significantly lower in both resting and c-myc-overexpressing fibroblasts. Accordingly, the c-Myc-induced transformation and clonal growth in the semiliquid agarose were completely ineffective in the HO-1-deficient cells, which can support the observation of reduced malignant transformation in the HO-1 knockout mice. Furthermore, the lack of HO-1 reduced migration capabilities of fibroblasts. A significant positive role for HO-1 in cell motility was earlier observed in endothelial progenitor cells [55] and keratinocytes [56]. Thus, one can suppose that reduced motility of cells with a low HO-1 expression may be associated with the lower infiltration of tissues and less malignant phenotype of cells.

In summary, DMBA/PMA treatment of HO-1 knockout mice induced the formation of skin lesions at the earliest, and resulting tumors grew the biggest, which was accompanied by increased mortality of the animals. However, histological analysis showed that the tumors remained benign papillomas. In contrast, tumors in the wild-type mice were smaller and developed later, but much more effectively underwent malignant conversion. Heterozygous animals grew relatively small, but very numerous, dysplastic tumors. Thus, HO-1 is protective against cancer initiation, but then seems to facilitate clonal promotion. These data also suggest that patients with a low expression of HO-1 may be more susceptible to development of SSC after exposure to carcinogens.

The following are the supplementary materials related to this article.
